# The Origin of Additive Genetic Variance Driven by Positive Selection

**DOI:** 10.1093/molbev/msaa085

**Published:** 2020-04-03

**Authors:** Li Liu, Yayu Wang, Di Zhang, Zhuoxin Chen, Xiaoshu Chen, Zhijian Su, Xionglei He

**Affiliations:** m1 State Key Laboratory of Biocontrol, School of Life Sciences, Sun Yat-Sen University, Guangzhou, China; m2 Zhongshan School of Medicine, Sun Yat-Sen University, Guangzhou, China; m3 Department of Cell Biology, Jinan University, Guangzhou, China

**Keywords:** adaptive divergence, population admixture, complex trait, additive variance, Fisher’s theorem of natural selection, positive selection

## Abstract

Fisher’s fundamental theorem of natural selection predicts no additive variance of fitness in a natural population. Consistently, studies in a variety of wild populations show virtually no narrow-sense heritability (*h*^2^) for traits important to fitness. However, counterexamples are occasionally reported, calling for a deeper understanding on the evolution of additive variance. In this study, we propose adaptive divergence followed by population admixture as a source of the additive genetic variance of evolutionarily important traits. We experimentally tested the hypothesis by examining a panel of ∼1,000 yeast segregants produced by a hybrid of two yeast strains that experienced adaptive divergence. We measured >400 yeast cell morphological traits and found a strong positive correlation between *h*^2^ and evolutionary importance. Because adaptive divergence followed by population admixture could happen constantly, particularly in species with wide geographic distribution and strong migratory capacity (e.g., humans), the finding reconciles the observation of abundant additive variances in evolutionarily important traits with Fisher’s fundamental theorem of natural selection. Importantly, the revealed role of positive selection in promoting rather than depleting additive variance suggests a simple explanation for why additive genetic variance can be dominant in a population despite the ubiquitous between-gene epistasis observed in functional assays.

## Introduction

An intriguing issue in genetics is how the additive genetic variance of a complex trait evolved in a population given that the epistasis between genes appears to be ubiquitous according to functional studies (Costanzo et al. 2010; Sackton and Hartl 2016). The Fisher’s fundamental theorem of natural selection predicts little additive variance (or narrow-sense heritability, *h*^2^) for fitness, because natural selection will fix alleles with the highest fitness quickly (Mousseau and Roff 1987; Merila and Sheldon 1999b; Crow 2002). An extended prediction of the theorem is that traits tightly coupled with fitness (i.e., evolutionarily important traits) should have smaller *h*^2^ than those less-coupled with fitness (Kruuk et al. 2000), because the response to natural selection on fitness will shape the evolution of the related traits (Orr 2009). The negative correlation between *h*^2^ and trait importance has been found in a variety of studies on different species or populations (Merila and Sheldon 1999a, 2000; Kruuk et al. 2000; Stirling et al. 2002; Teplitsky et al. 2009; Wheelwright et al. 2014; Sztepanacz et al. 2017). For example, for the wild female red deer (*Cervus elaphus*), the *h*^2^ of several life-history traits, including the total number of offsprings, the adult breeding success, and the longevity, are all zero (Kruuk et al. 2000). Meanwhile, the morphologic traits such as leg length and jaw length, which are believed to be less related to fitness, are found to have much higher *h*^2^ than the life-history traits. The pattern is also true for collared flycatcher (*Ficedula albicollis*), Savannah sparrows (*Passerculus sandwichensis*), red-billed gull (*Larus novaehollandiae*), and so on (Merila and Sheldon 2000; Stirling et al. 2002; Teplitsky et al. 2009; Wheelwright et al. 2014).

However, there are also reports of abudant additive variances for important traits (Pettay et al. 2005; Teplitsky et al. 2009; Kosova et al. 2010; Milot et al. 2011; Zhang 2012). In particular, there is sometimes even a positive correlation between *h*^2^ and trait importance. For example, in a bighorn sheep population from Ram Mountain, the lowest *h*^2^ was for body mass at primiparity (0.02), whereas the *h*^2^ of lifetime fecundity was as high as 0.66 (Reale and Festa-Bianchet 2000). A variety of explanations to the observations have been proposed. In addition to considering the different variance components of *h*^2^ (Visscher et al. 2008), or balancing selection (Barton and Keightley 2002; Grieshop and Arnqvist 2018), a predominant view is that fluctuating environments combined with mutations could help maintain high additive variance of fitness (Burger and Gimelfarb 2002; Crow 2008; Zhang 2012). These explanations are all theoretical, lacking empirical evidence. More importantly, they do not predict a positive correlation between *h*^2^ and trait importance.

We reason that here an ecological factor in evolution, namely, migration, may play an essential role. For a given species, there are often plenty of divergences between populations (Pizzo et al. 2008; Sved et al. 2008; Roy et al. 2014). When the divergences are coupled with local adaptation (i.e., adaptive divergences), which happens quite often in nature (Pulido 2007; Liedvogel et al. 2011), alleles with beneficial additive effects on important traits would be preferentially fixed in a population. As different genes would be selected for in different populations, subsequent population admixture by migration would lead to new populations with abundant additive genetic variances for important traits. In this study, we designed an experimental test for this reasonable hypothesis, revealing a birth–death cycle of additive variance driven by positive selection.

## Results

**Fig. 1.**
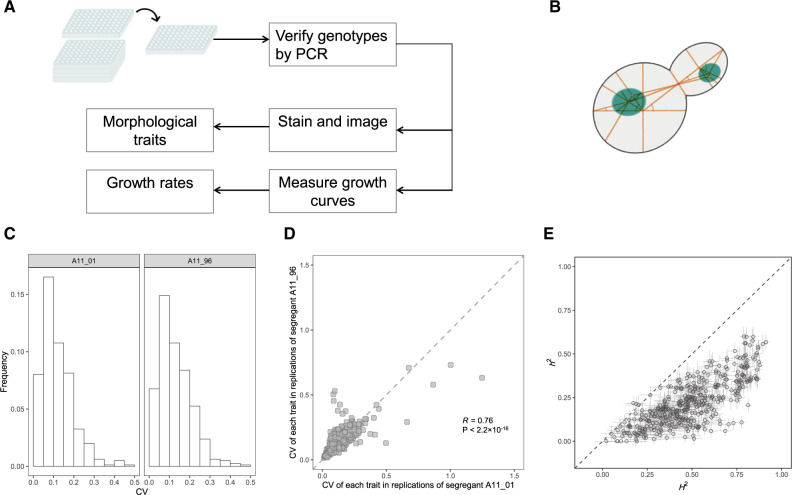
Measuring 405 cell morphological traits and their heritability in the yeast segregant population. (*A*) The experimental process in this study. Both morphological traits and growth rate of each segregant were measured with replications. (*B*) The schematic diagram of a yeast cell with basic coordinates for characterizing cell morphological traits. (*C*) The distribution of CV of the 405 traits calculated from technical replicates of segregants A11_01 (left) and A11_96 (right), respectively. (*D*) The CVs obtained from segregants A11_01 and A11_96 are consistent (Pearson’s *R* = 0.76, *P* < 2.2 × 10^−16^, *N* = 405). (*E*) The broad-sense heritability (*H*^2^) and narrow-sense heritability (*h*^2^) of each trait. Error bars represent SE.

We examined a panel consisting of ∼1,000 prototrophic haploid yeast segregants produced from a cross of two *Saccharomyces cerevisiae* strains (BY parent and RM parent). The two parental strains differ by ∼0.5% at the genomic sequence level and experienced adaptive divergence according to an analysis of a set of principle component traits (Ho et al. 2017). The ∼1,000 segregants were all genotyped in a previous study (Bloom et al. 2013). We first verified the segregant panel and removed the segregants that appeared to be discordant with the reported genotypes (fig. 1*A*; see Materials and Methods).

We measured 405 cell morphological traits for each segregant with two technical replications by following a previous protocol with some modifications (see Materials and Methods) (Ohya et al. 2005). These traits are related to the characters of mother cell and/or bud in different stages, such as area, distance, localization, angle, ratio, and so on (fig. 1*B*). After excluding measurements with insufficient cell number for calculating traits, we obtained the morphological trait information for 734 segregants, 73.3% (538/734) of which had data of at least two replications (supplementary table S1, Supplementary Material online). Approximately 99.5% of the trait values were derived from >100 cells of a given segregant (supplementary fig. S1, Supplementary Material online). Segregants A11_01 and A11_96 were measured in every experiment as a technical control for potential operating bias in culturing, staining, and imaging. We calculated the measuring coefficient of variance (CV) of each morphological trait using the 26 technical replications of segregant A11_01, or the 28 technical replications of segregant A11_96, respectively (see Materials and Methods). The obtained CVs were generally small, with >80% of them being <0.2 (347/405 in A11_01 and 326/405 in A11_96; fig. 1*C*). In addition, they were consistent between measures in segregant A11_01 and in A11_96 (Pearson’s *R *=* *0.76, *P *<* *2.2 × 10^−16^; fig. 1*D*). These data together suggested no strong batch effects in the trait measurements. We also checked pairwise rank correlation of the 405 traits between 28 technical replications of the segregant A11_96, or 26 replications of the segregant A11_01, or two replications of 536 segregants, respectively. We observed invariably strong correlations (supplementary fig. S2, Supplementary Material online). The large number of high-quality quantitative traits of the same property (i.e., morphology) measured under the same experimental setting provided a unique opportunity to study the evolution of additive genetic variance.

Quantile normalization of the raw trait values was performed to ensure the different traits comparable (see Materials and Methods). The broad-sense heritability *H*^2^ and narrow-sense heritability *h*^2^ were estimated for each of the 405 traits according to a previous study (Bloom et al. 2013) (supplementary table S2, Supplementary Material online). The *H*^2^ of the 405 traits ranged from 0.021 to 0.913, with a median of 0.478; the *h*^2^ ranged from 0.000 to 0.619, with a median of 0.240 (fig. 1*E*). In this study, there were no dominance effects because the segregants are haploid; the gene–environment interactions should be weak because the same culture condition was used. Thus, here, *H*^2^ is the proportion of phenotypic variance (*V*_P_) explained by additive (*V*_A_) and nonadditive (or epistatic) effects (*V*_Non-A_), and *h*^2^ = *V*_A_/*V*_P_. Because normalized trait values were considered, *V*_P_ of the different traits was within an ∼1.5-fold range, whereas *h*^2^ spanned a >100-fold range (supplementary fig. S3, Supplementary Material online). As a result, in this study, *h*^2^ served effectively as a direct measure of *V*_A_ because they were highly correlated with Pearson’s *R *=* *0.99 among the 405 traits (supplementary fig. S3, Supplementary Material online).

**Fig. 2.**
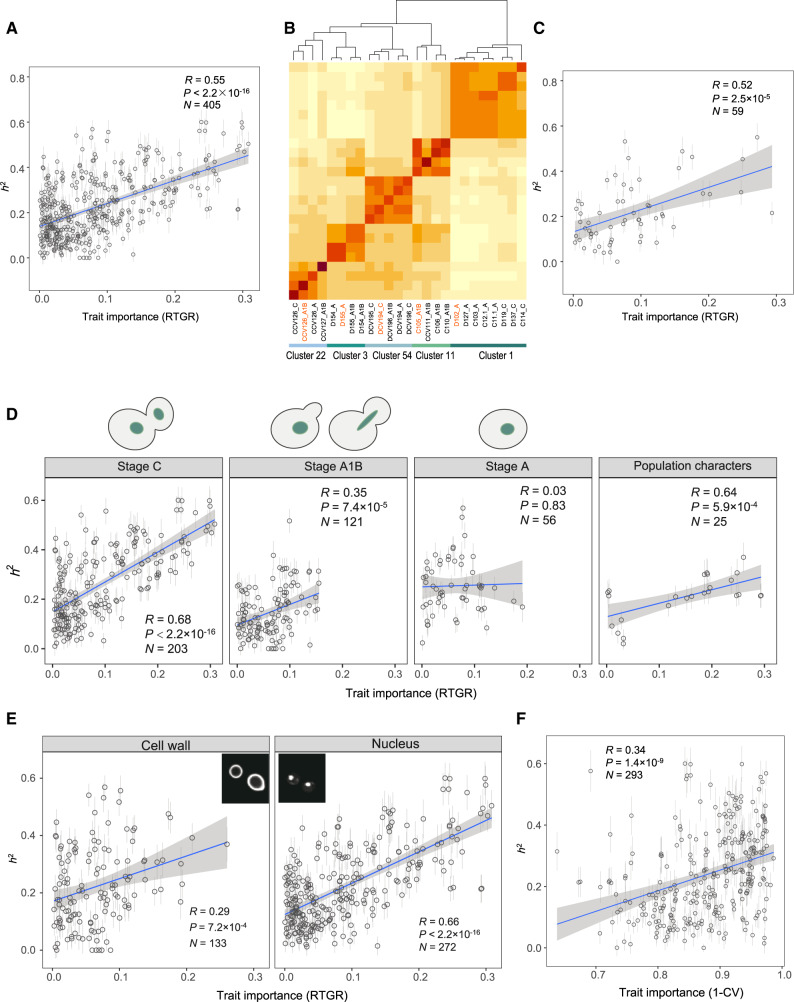
Evolutionarily important traits tend to have a large *h*^2^ in the segregant population. (*A*) A strong positive correlation between *h*^2^ and trait importance measured by RTGR (Pearson’s *R* = 0.55, *P* < 2.2 × 10^−16^, *N* = 405). Error bars represent SE. The gray zone shows 95% confidence interval of the regression line. (*B*) Define 59 exemplary traits from the 405 traits. The heatmap shows trait similarity in five randomly chosen clusters derived by apcluster, with one exemplary trait highlighted in orange from each cluster. (*C*) The correlation between *h*^2^ and RTGR remains for exemplary traits (Pearson’s *R* = 0.52, *P* = 2.5 × 10^−5^, *N*= 59). (*D*) The correlation between *h*^2^ and RTGR largely holds for traits characterized at different stages. The top diagrams show the states of a yeast cell that separate cell stages. (*E*) The correlation between *h*^2^ and RTGR remains for traits related to cell wall (stained by FITC-ConA) or to nucleus (stained by Hoechst). (*F*) A positive correlation between *h*^2^ and trait importance measured by 1−CV (Pearson’s *R* = 0.34, *P* = 1.4 × 10^−9^, *N* = 293).

To assess the evolutionary importance of the morphological traits, we computed their relatedness to growth rate (RTGR). We measured the growth rate of each segregant under the same condition as for trait measurement (fig. 1*A* and supplementary table S3, Supplementary Material online). For each of the 405 traits, we computed the Pearson’s correlation coefficient (Pearson’s *R*) between trait value and cell growth rate among the 734 segregants. Following a previous study (Chen et al. 2018), the absolute value of Pearson’s *R* was then used as the RTGR of a morphological trait; traits with larger RTGR are regarded as evolutionarily more important. The value of RTGR varied from 0 to 0.308, with a median of 0.065, highlighting a wide range of evolutionary importance of the 405 morphological traits (fig. 2*A*).

According to our hypothesis, the admixture of two populations with adaptive divergence would result in a new population with more additive variances in evolutionarily more important traits. The availability of both *h*^2^ and evolutionary importance for the large number of traits enabled a direct test for the hypothesis. Consistent with the hypothesis, we found a strong positive correlation between *h*^2^ and trait importance estimated by RTGR among the 405 yeast traits (Pearson’s *R *=* *0.55, *P *<* *2.2 × 10^−16^; fig. 2*A*). Because many traits are correlated with each other, we conducted affinity propagation clustering and obtained 59 trait clusters each with an exemplary trait (fig. 2*B* and supplementary fig. S4, Supplementary Material online). The number of traits represented by an exemplary trait ranged from 2 to 16, with a median of 6, and there were only weak correlations among the 59 exemplary traits (supplementary fig. S4, Supplementary Material online). The strong positive correlation between *h*^2^ and RTGR remained when only the exemplary traits were considered (Pearson’s *R *=* *0.52, *P *=* *2.5 × 10^−5^, fig. 2*C*).

The 405 traits represent cell morphology at different cell cycle stages. We divided these traits into four categories according to the states of bud and nucleus (see Materials and Methods). Traits of stage A1B and stage A tended to have small RTGR, suggesting less selective constraints on the morphology of the two stages. Importantly, the positive correlation between *h*^2^ and RTGR remained with the exception for traits of stage A (fig. 2*D*). In addition, as the 405 traits represent features of cell wall and nucleus that are stained by two different dyes FITC-ConA and Hoechst, respectively (see Materials and Methods), we examined the 133 cell wall-related traits and 272 nucleus-related traits separately. The positive correlation between *h*^2^ and RTGR held in both categories (fig. 2*E*).

A previous study suggests the CV in trait measurement could serve as a proxy of trait importance, with smaller CV for more important traits (Ho and Zhang 2014). To be conservative we considered only 293 traits that have consistent CV between A11_01 and A11_96, and used the average to represent trait importance (supplementary fig. S5, Supplementary Material online). To be consistent with RTGR in the direction of trait importance, we considered 1−CV rather than CV. We observed a positive correlation between *h*^2^ and 1−CV (Pearson’s *R *=* *0.34, *P *=* *1.4 × 10^−9^; fig. 2*F*), a result supporting our hypothesis. The pattern generally remained by considering exemplary traits or by separating the traits into different categories (supplementary fig. S5, Supplementary Material online).

**Fig. 3.**
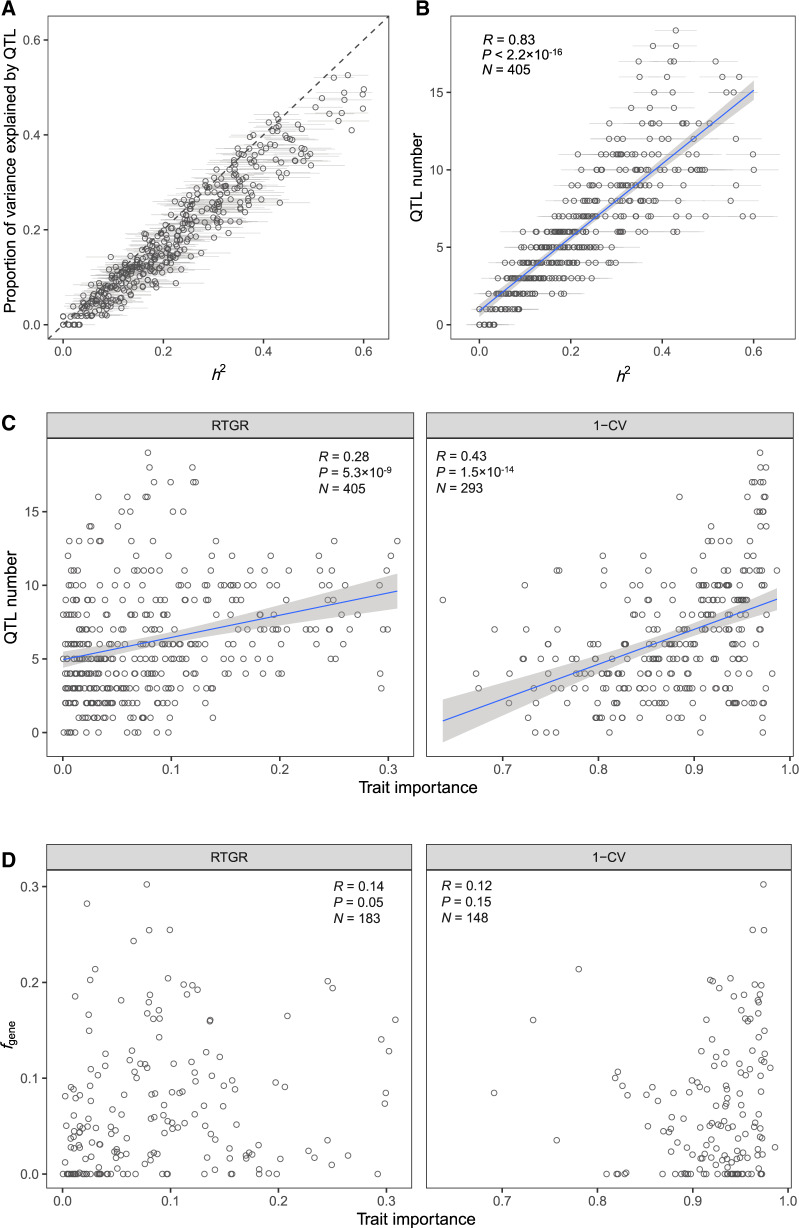
Analysis of QTLs of the 405 traits. (*A*) The proportion of phenotypic variance explained by QTLs is close to *h*^2^, suggesting a nearly saturation of the QTL detection. Error bars represent SE. (*B*) A strong positive correlation between QTL number and *h*^2^ (Pearson’s *R* = 0.83, *P* < 2.2 × 10^−16^, *N* = 405). The gray zone shows 95% confidence interval of the regression line. (*C*) A moderately positive correlation between QTL number and trait importance measured by RTGR (left) or 1−CV (right). (*D*) No apparent correlation between the fraction of genes that affect a trait (*f*_gene_) and trait importance. Only those traits examined in panel C have estimated *f*_gene_ values are included.

We then mapped quantitative trait loci (QTL) for each of the traits. A total of 2,505 QTLs were detected for 393 traits (supplementary table S4, Supplementary Material online), and the number of QTLs ranged from 1 to 19, with a median of 6 for each trait (supplementary fig. S6, Supplementary Material online). There were 12 traits with no detectable QTLs, which conforms to their extremely low *h*^2^ (median *h*^2^ ∼ 0.016). In nearly all cases, the trait variance explained by detected QTLs was close to *h*^2^ (Pearson’s *R *=* *0.96, *P *<* *2.2 × 10^−16^, fig. 3*A*), suggesting a nearly saturation of the QTL detection. This is consistent with a previous observation in the yeast segregant panel (Bloom et al. 2015). Most of the QTLs (∼90.6%) each explained a small proportion (<5%) of the trait variance (supplementary fig. S6, Supplementary Material online).

We found the *h*^2^ of a trait was highly correlated with the number of QTLs (Pearson’s *R *=* *0.83, *P *<* *2.2 × 10^−16^; fig. 3*B*). Consistently, there were in general more QTLs detected for more important traits (fig. 3*C* and supplementary fig. S7, Supplementary Material online). This result indicated there are more diverged loci for important traits after the split of the two parental yeasts of the segregant population examined here. There are two possible explanations: First, there are more genes and thus more mutations that affect important traits; second, there are higher fixation rates for mutations that affect important traits. To distinguish them, we examined the cell morphology data generated for a large set of yeast single gene deletion mutants. For each of the traits, we obtained the fraction of genes that affect a trait (*f*_gene_) by following a previous study (Ho and Zhang 2014). We failed to observe a larger *f*_gene_ for more important traits (fig. 3*D*), suggesting the second explanation is plausible although the number of whole genes affecting a trait does not necessarily tell the number of natural variants affecting the trait. A higher fixation rate of mutations affecting more important traits indicates positive selection underlies the genetic divergence of the parental yeasts. This echoes the adaptive phenotypic evolution of the yeast *S. cerevisiae* previously proposed based on the faster phenotypic evolution of more important traits (Ho et al. 2017). Of note, the many QTLs detected for an important trait often showed opposite effects in a parent (supplementary fig. S8, Supplementary Material online), indicating the phenotypic divergence between the two parents does not represent well the underlying genetic divergence. This may explain the relatively weak signal of adaptive phenotypic divergence between the two parents (Ho et al. 2017).

## Discussion

Fisher’s fundamental theorem of natural selection provides a general framework for thinking of the evolution of additive genetic variance. Previous empirical studies on this issue are all based on wild populations and the resulting patterns are discordant, which are often ascribed to confounding ecological factors. This study is, to the best of our knowledge, the first controlled experiment for examining the relationship between additive variance and evolutionary importance in a large set of quantitative traits. The advantage of controlled experiments is the ecological variables in wild populations, such as nutrition, parasite, predator, and so on, are all fixed. However, there is a caveat in our experiment. Specifically, as the proposed adaptive divergence of the two parental yeasts must occur in natural environments, the trait importance obtained in the lab condition may not necessarily represent that of the natural environments. Nevertheless, this problem would most likely reduce the correlation between *h*^2^ and trait importance, underestimating the contribution of positive selection to the origin of additive variance.

**Fig. 4.**
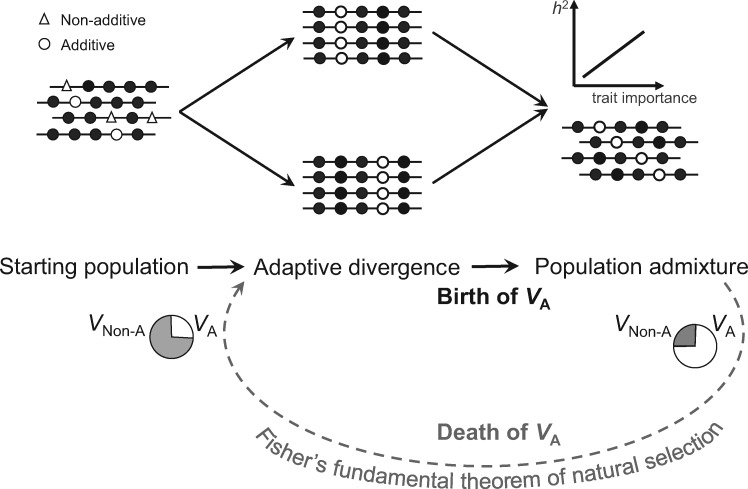
A birth–death cycle of additive variance driven by positive selection. The birth process is explained by adaptive divergence followed by population admixture. During adaptive divergence, different genetic loci would be positively selected in the diverged populations. Although nonadditive variances (*V*_Non-A_) could be the majority in the starting population, additive alleles but not nonadditive alleles would be preferentially fixed. The subsequent population admixture would then result in a new population full of additive variances (*V*_A_). The death of additive variances is a typical process described by Fisher’s fundamental theorem of natural selection. By assuming frequent adaptive divergences followed by population admixture, there could be a constantly active birth–death cycle underlying the structure of additive variances of a species.

The unexpected role positive selection could play in promoting additive variance provides an solution to a long-standing puzzle, namely, that the additive variances are often pervasive in a population despite the ubiquitous nonadditive (or epistatic) interactions observed between genes in functional studies (Costanzo et al. 2010; Sackton and Hartl 2016). A previous explanation to the puzzle considers the variance allele frequencies that are often J- or U-shaped distribution in natural populations, which minimizes epistasis by precluding multilocus genotypes (Hill et al. 2008). This, however, cannot explain why *h*^2^ is comparably large in experimental populations with uniform allele frequencies of ∼50% (Bloom et al. 2013). In the model of adaptive divergence followed by population admixture, the process of adaptive divergence serves effectively as a filter to remove nonadditive alleles that could be the majority of raw mutations. This is because positive selection favors the fixation of additive alleles but not nonadditive alleles. The subsequent population admixture would then result in a population full of additive variances (fig. 4).

Notably, the acquired additive variances would be depleted also by selection in a way clearly described by Fisher’s fundamental theorem of natural selection. We conducted simulations to track the depletion process for a representative trait with an *h*^2^ ∼ 0.6 in the yeast segregant population, and observed a rapid reduction of *h*^2^ (supplementary fig. S9, Supplementary Material online). Hence, there is a birth–death cycle of additive variance driven by positive selection, which could be repeated again and again by assuming pervasive adaptive divergences followed by population admixtures in some species (fig. 4). Populations at different stages of the cycle would have different structures of additive variance. Under the Wright–Fisher model with natural selection, it would take ∼1,000 (or ∼10,000) generations for a new beneficial mutation with *s *=* *0.01 (or = 0.001) to be fixed in a diploid population of *N *=* *100,000, where *N* is the population size and *s* is the selection coefficient (Otto and Whitlock 2013). The time will be shorter for alleles with larger than 1/2 *N* initial frequency, which is the case for populations resulted from admixture (Kimura 1983). These numbers are useful for thinking of the time scale of the birth and death of an additive variance in the cycle.

The proposed origin of additive variance in this study is of particular relevance to the following scenarios. First, our knowledge of quantitative genetics is often from studies on laboratory populations that are produced by crossing two or several strains/lines of a model organism such as yeast or fruit fly (Bloom et al. 2013; Long et al. 2014). As in the current study, the additive variances of these populations are all explained by the birth process of variance. Second, in the breeding of crops or livestock there are often a few to a few ten generations (Wiener et al. 1992; Hinze and Lamkey 2003). As a result, the additive variance structure of a breeding population should be also dominated by the birth process. Third, our human beings have both wide geographic distribution and strong migratory capacity, the former predicting frequent local adaptions (i.e., adaptive divergences) and the latter enabling repeated population admixtures (Hellenthal et al. 2014; Fu et al. 2016). Hence, the selection-driven birth–death cycle of additive variance could have been constantly active during the human evolution. The resulting additive variance structure in current human populations determines how human complex traits can be studied and understood.

## Materials and Methods

### Verify Segregant Panel

The segregant panel was kindly provided by Dr L. Kruglyak. There were total 1,056 segregants in eleven 96-well plates. To verify the genotypes, 12 segregants in each plate were randomly picked up and four loci (MATa, MATα, hphMX4, natMX4) were amplified by polymerase chain reaction for these segregants. By comparing the results with the genotypes provided by Dr L. Kruglyak, we found that some percentage of segregants in plates 8 and 9 were mismatched, and there was no pattern to rescue the segregants in a row or a line, which may be the result of contaminations. We then focused the segregants in the left nine plates with right genotypes in the next experiments.

### Measure Cell Morphological Traits

The morphological traits of each segregant were measured following Ohya’s protocol with some modifications (Ohya et al. 2005). Briefly, segregants were grown in YPD medium (yeast extract/peptone/dextrose medium) to saturation phase at 25 °C for 2 or 3 days, and then transferred to new cultures to exponential phase at 25 °C for 3 or 4 h. Each segregant had two replications. Cells were fixed with 3.7% formaldehyde solution. Cell walls were stained with FITC-ConA (fluorescein isothiocyanate-conjugated, concanavalin A, Sigma–Aldrich C7642). Cell nucleus was stained by Hoechst-mix (Thermo Fisher, Hoechst 33342 Solution) instead of DAPI to enhance the specificity. We omitted the process of actin staining because the dye of actin (Rhodamine phalloidin) was not stable and could not support to image for a long time in the high-throughput automated image-processing. The stained cells were plated on microplates (Greiner 781091) with ∼5.0 × 10^4^ cells per well and taken images by IN Cell Analyzer 2200 (GE Healthcare) with 100× objective lens. There were two technical replications for each segregant, and segregants A11_01 and A11_96 were cultured, stained, and imaged in every experiment as a technical control.

CalMorph software was used to analyze images to quantify yeast morphology, and 405 quantitative traits were derived (Ohya et al. 2005). Segregants whose cell number for calculating traits <80 in both two replications were excluded. Values of all traits were listed in supplementary table S1, Supplementary Material online. There were 734 segregants each with 405 morphological traits derived, in which 73.3% (538/734) had at least two replications. Quantile normalization was performed to the raw values of traits by R package preprocessCore for further calculations (https://github.com/bmbolstad/preprocessCore).

Traits derived from cell wall or nucleus can be distinguished by the initial letter of traits, in which “C” is related to cell wall and “D” is related to nucleus. Traits in different stages can be distinguished by the letters after the connector line. “A” represents traits calculated by cells with one nucleus and without a bud, “A1B” is traits calculated by cells with one nucleus in the mother cell with a bud or the nucleus is dividing at the neck, and “C” is traits derived by cells with one nucleus each in the mother cell and bud. The 405 traits are not independent, and 59 exemplary traits were derived by R package “apcluster” (negDistMat, *r* = 2) using the mean normalized values of 734 segregants (Frey and Dueck 2007).

### Measure Cell Growth Rate

Strains were grown in YPD medium to saturation phase at 25 °C for 2 or 3 days, then diluted 1:100 to 100 μl fresh YPD medium at 96-well plate. Two replications of each segregant were placed in the same 96-well plate. The 96-well plates were put on Epoch2 Microplate Spectrophotometer (BioTek) and incubated at 25 °C with shaking. The absorbances at 600 nm of each well were determined per hour. The measurements lasted 24 h and all strains reached saturation phase. The *V*_max_ of growth rate, that is, the maximum slope of growth curve of each well, was used to estimate the fitness of each strain. To control the positional bias, the original values of growth rate in each plate were fit by a robust locally weighted regression by R package “locfit” according to Bloom et al.’s study (2013). The average normalized values of growth rate were taken as the fitness of each segregant, and listed in supplementary table S3, Supplementary Material online.

### Calculate Heritability

Because the segregant panel was produced by Bloom et al., broad-sense heritability (*H*^2^), narrow-sense heritability (*h*^2^), additive QTL, and the variance explained by QTL of each morphological trait were calculated by methods consisted with Bloom et al.’s study (2013). Briefly, *H*^2^ was calculated by normalized values of traits of segregants with two replications. *H*^2^ was estimated as $\sigma_{G}^{2}/{(\sigma }_{G}^{2}+\sigma_{E}^{2})$, where $\sigma_{G}^{2}$ was the genetic variance and $\sigma_{E}^{2}$ was the error variance. It was performed by the “lmer” function in lme4 R package (Bates et al. 2015). When compared with *H*^2^, *h*^2^ was calculated by the average normalized values of traits of segregants with two replications. And, segregants with only one replication were also included in other situations. Narrow-sense heritability was estimated as $\sigma_{A}^{2}/{(\sigma }_{A}^{2}+\sigma_{\mathrm{EV}}^{2})$, where $\sigma_{A}^{2}$ was the additive genetic variance and $\sigma_{\mathrm{EV}}^{2}$ was the error variance. R package rrBLUP was used to calculate *h*^2^ (Endelman 2011). SEs of *H*^2^ and *h*^2^ were calculated by delete-one jackknife both.

Additive QTL of each trait was detected using the stepwise forward-search approach developed by Bloom et al. (2013). LOD scores for each genotypic marker and each trait were calculated as $-n\left(\frac{\ln\left(1-r^{2}\right)}{2\ln\left(10\right)}\right)$, where *r* is the Pearson correlation coefficient between the genotypes and trait values. Significant genetic markers were detected from four rounds using different LOD thresholds corresponding to a 5% FDR, which were 2.68, 2.92, 3.72, and 4.9, respectively. A multiple regression linear model was estimated by taken each QTL as independent variables of each trait, and the total phenotypic variance explained by additive QTL was the square of the multiple regression coefficient. The results were listed in supplementary table S2, Supplementary Material online.

### Calculate Trait RTGR

For each trait, the average normalized values of replications of each segregant were calculated. For segregants with only one replication, the trait values were the normalized values from the only measurement. The absolute Pearson’s *R* between the trait values and the growth rates in YPD medium across 734 segregants was used as a proxy of RTGR for each trait. The results were listed in supplementary table S2, Supplementary Material online.

### Calculate Trait CV among Replications

Coefficient of variations for each trait was calculated using raw data of replications of A11_01 and A11_96, respectively. Traits with large CV may indicate their indeterminacy, so we excluded seven traits with CV >0.4 when using CV as an index of trait importance. To evaluate the repeatability of two groups, we used a distance index between two groups of CV as $\left|{\mathrm{CV}}_{01_{i}}-{\mathrm{CV}}_{96_{i}}\right|/{(\mathrm{CV}}_{01_{i}}+{\mathrm{CV}}_{96_{i}})$, where ${\mathrm{CV}}_{01_{i}}$ and ${\mathrm{CV}}_{96_{i}}$ were the values of CV for trait *i* in A11_01 and A11_96, respectively. A large CV distance means the environmental robustness of the trait would be different for different segregants, so we excluded 105 traits with CV distance index >0.2 to obtain a set of traits with consistent measurements. There were 293 traits left. The results were listed in supplementary table S2, Supplementary Material online. 

## Supplementary Material

msaa085_Supplementary_Data
