# Supplementary material for: The Origin of Additive Genetic Variance Driven by Positive Selection
Source: Mol Biol Evol. 2020 Apr 3;37(8):2300–8. doi: 10.1093/molbev/msaa085 (PMC7403624; doi:10.1093/molbev/msaa085)
Supplement: msaa085_Supplementary_Data [file msaa085_supplementary_data.zip › msaa085-suppl_data/MS-SI.pdf]

Supplementary Information of

"The origin of additive genetic variance driven by positive selection"

Li Liu[1#], Yayu Wang[1#], Di Zhang[1], Zhuoxin Chen[1], Xiaoshu Chen[2], Zhijian Su[3*] and Xionglei He[1*]

[1]State Key Laboratory of Biocontrol, School of Life Sciences, Sun Yat-Sen University, Guangzhou, 510275, China
[2]Zhongshan School of Medicine, Sun Yat-Sen University, Guangzhou, 510080, China
[3]Department of Cell biology, Jinan University, Guangzhou, 510632, China
[#]Equal contribution
*Correspondence should be addressed to X. H. (hexiongl@mail.sysu.edu.cn) or Z. S. (tjnuszj@jnu.edu.cn).

This file contains:
Legends for Tables S1-S4
Fig. S1-S9

Legends for supplementary tables
Table S1. Raw trait values of 734 segregants examined in this study.
Table S2. Summary of the analyses of heritability and trait importance.
Table S3. Average value of normalized growth rate of each segregant.
Table S4. Summary of the detected QTLs for each trait.

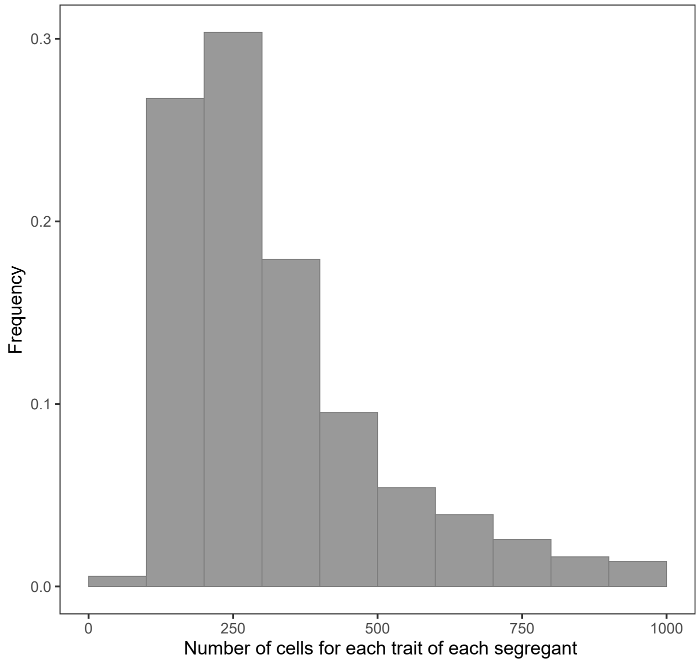

Figure S1. The distribution of cell numbers used to calculate each trait in each segregant.

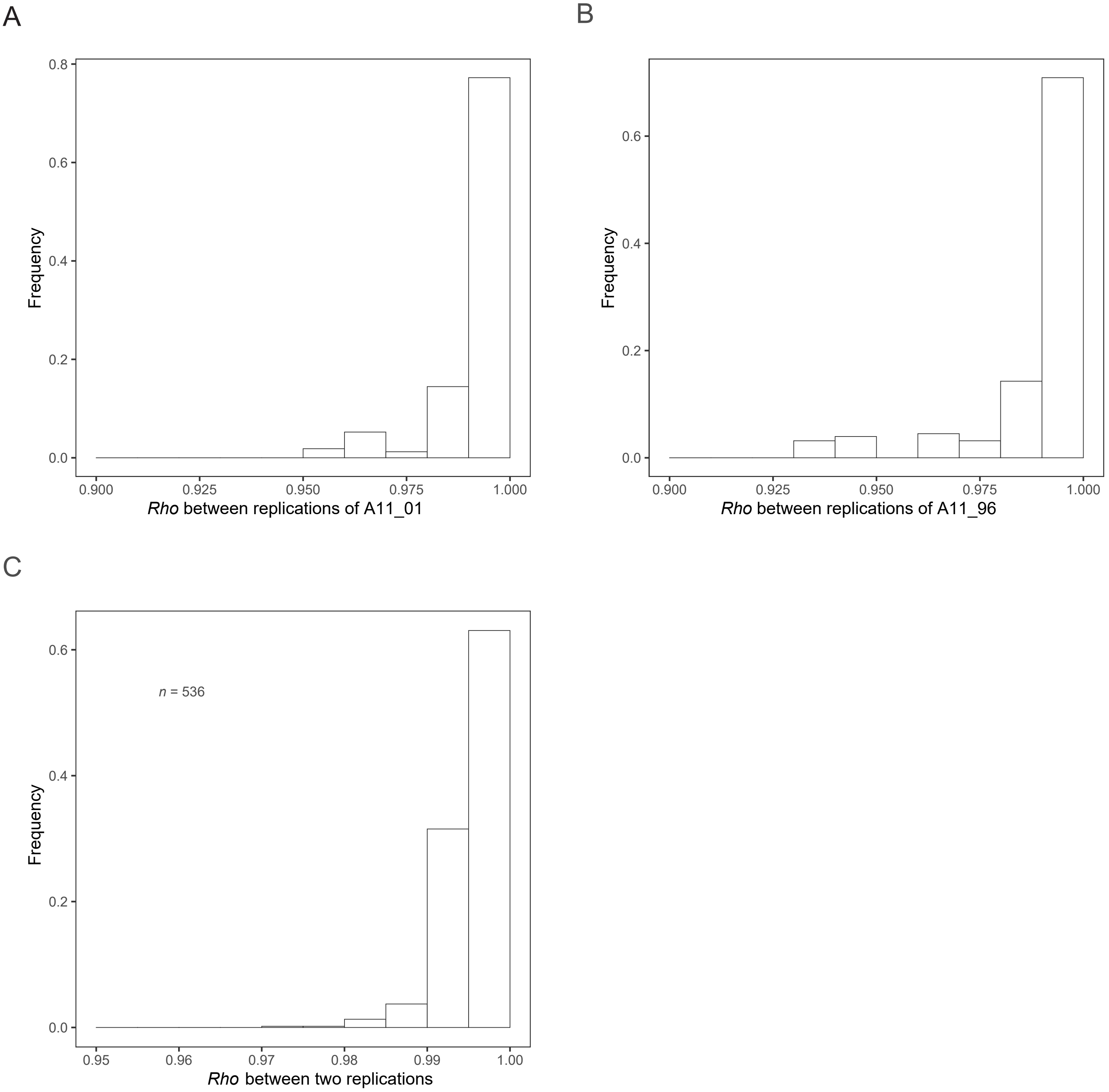

Figure S2. The distribution of correlations of the 405 traits between different replications of segregant A11_01 (A), segregant A11_96 (B), and the 536 segregants with two replications (C). Because the trait values span a wide range and are not directly comparable, Spearman's rank correlation analysis was conducted with the correlation coefficient *Rho* being shown.

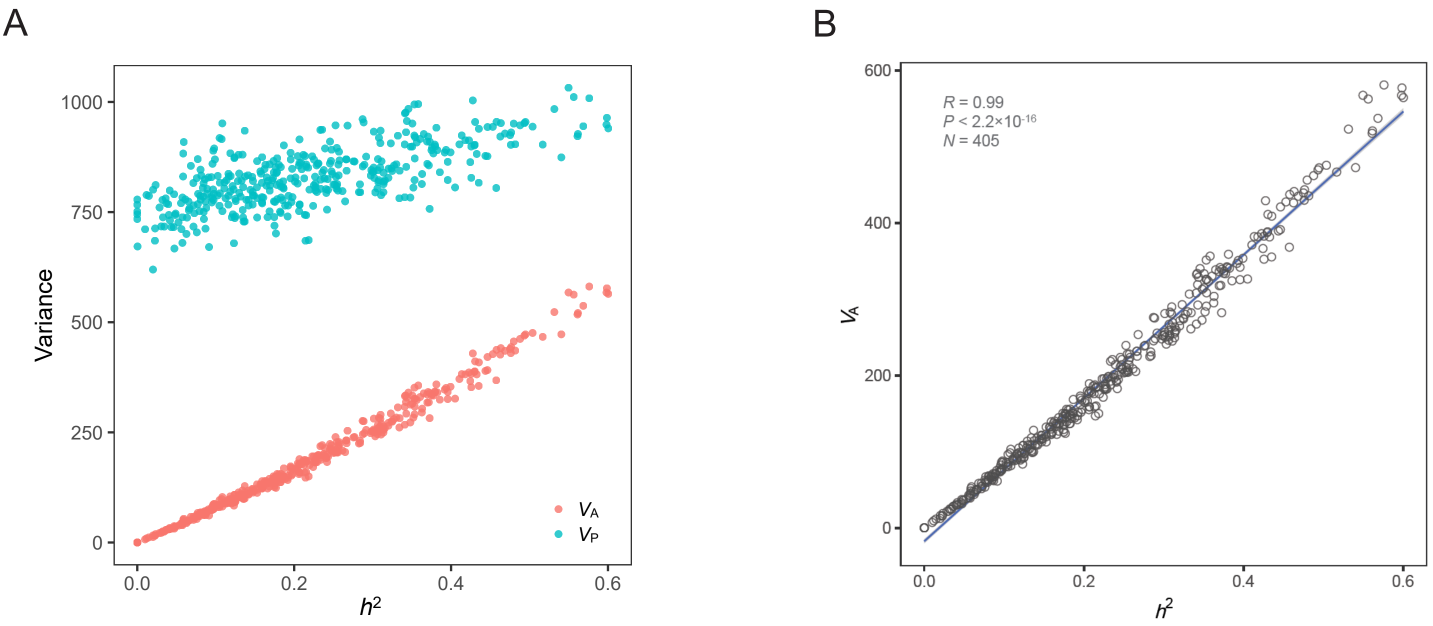

Figure S3. (A) V_P and V_A of the 405 traits, with the former spanning a narrow range but the latter a much wider range. (B) h^2 is nearly all explained by V_A (Pearson's $R = 0.99$, $P < 2.2\times10^{-16}$, $N = 405$).

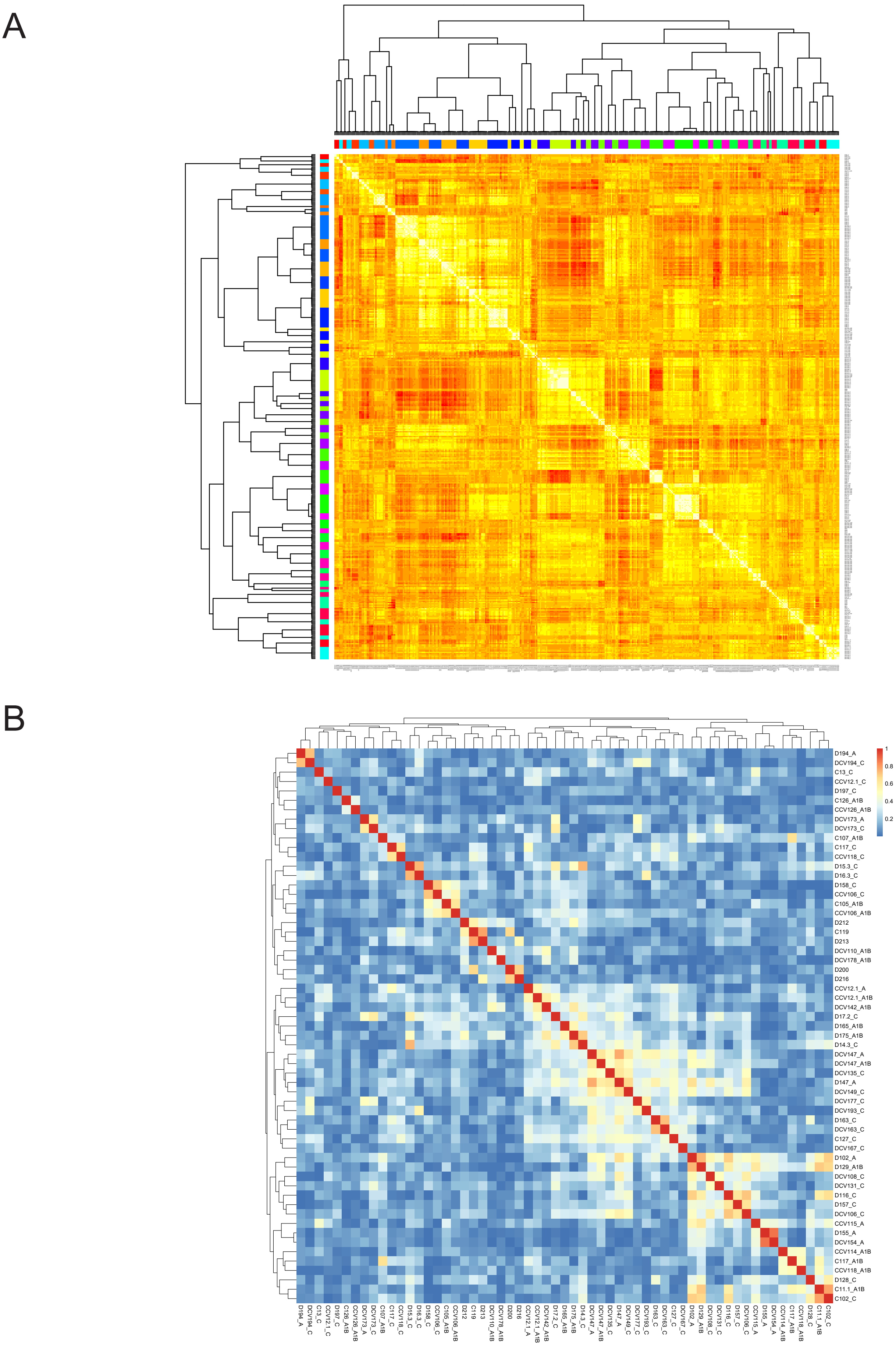

Figure S4. Define 59 exemplary traits from the 405 traits. (A) Clustering analysis of the 405 traits derivedby apcluster. (B) The pairwise correlations between 59 exemplary traits in the segregants.

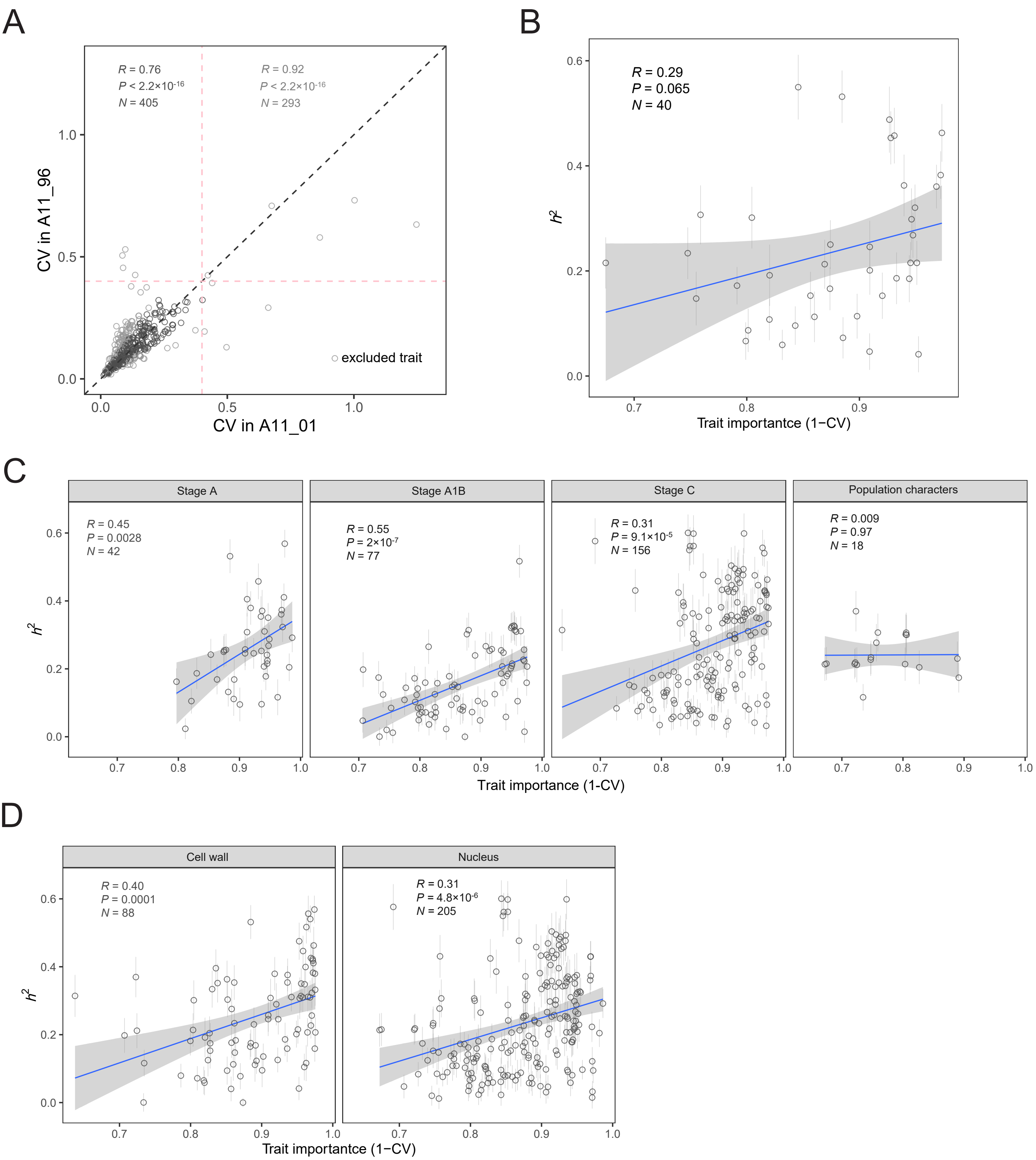

Figure S5. The correlation between h^2 and trait importance measured by 1-CV.
(A): The CVs of the traits obtained from the two segregants (A11_01 v.s. A11_96). The light gray circles show 112 traits that were excluded from further analyses, because for each of them the mean CV is greater than 0.4 or the difference between the two segregants is greater than 0.2. The gray dashed line is $y = x$, and the pink dashed lines represent $x = 0.4$ and $y = 0.4$, respectively.
(B): A moderate but statistically insignificant correlation between h^2 and 1-CV for 40 exemplary traits.
(C): The positive correlation between h^2 and 1-CV largely holds for traits characterized at different stages.
(D): The positive correlation between h^2 and 1-CV largely remains for traits related to cell wall or to nucleus.

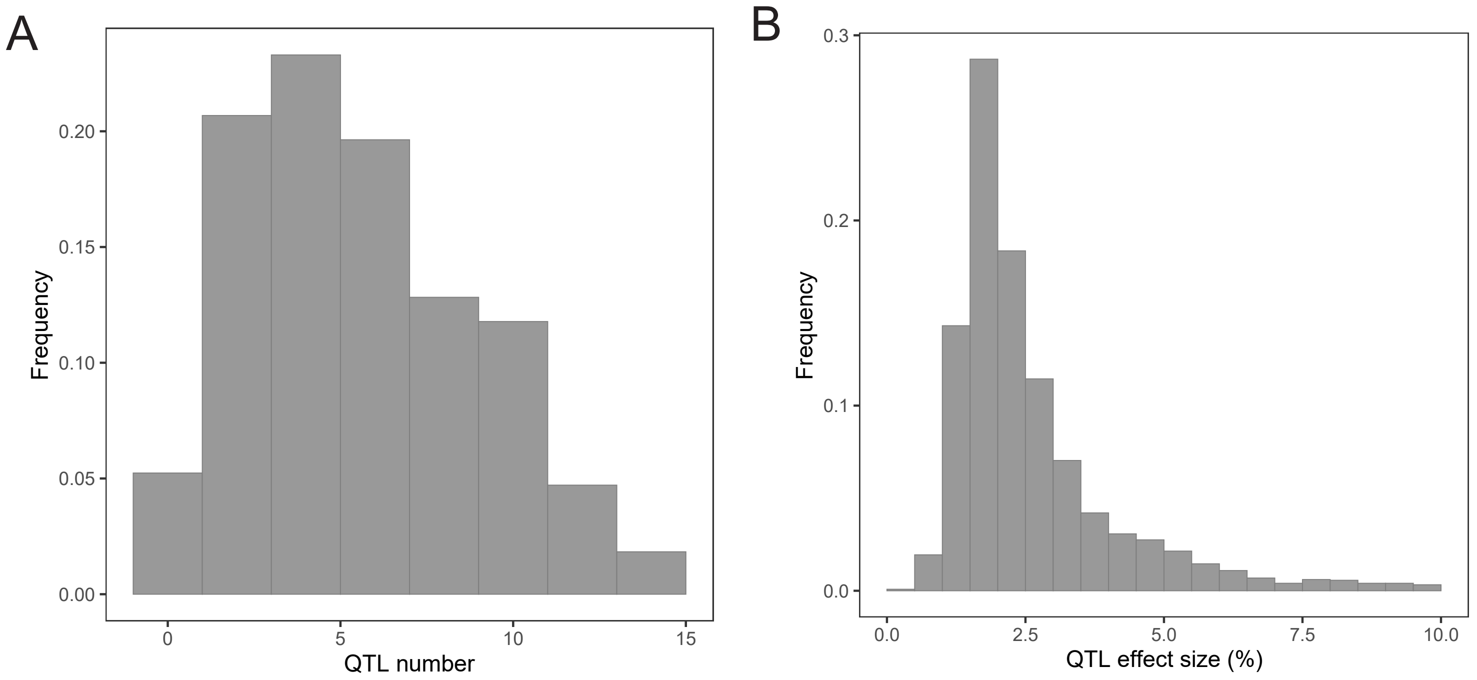

Figure S6. The distribution of QTL number (A) or per-QTL effect size (B) detected for each of the morphological traits.

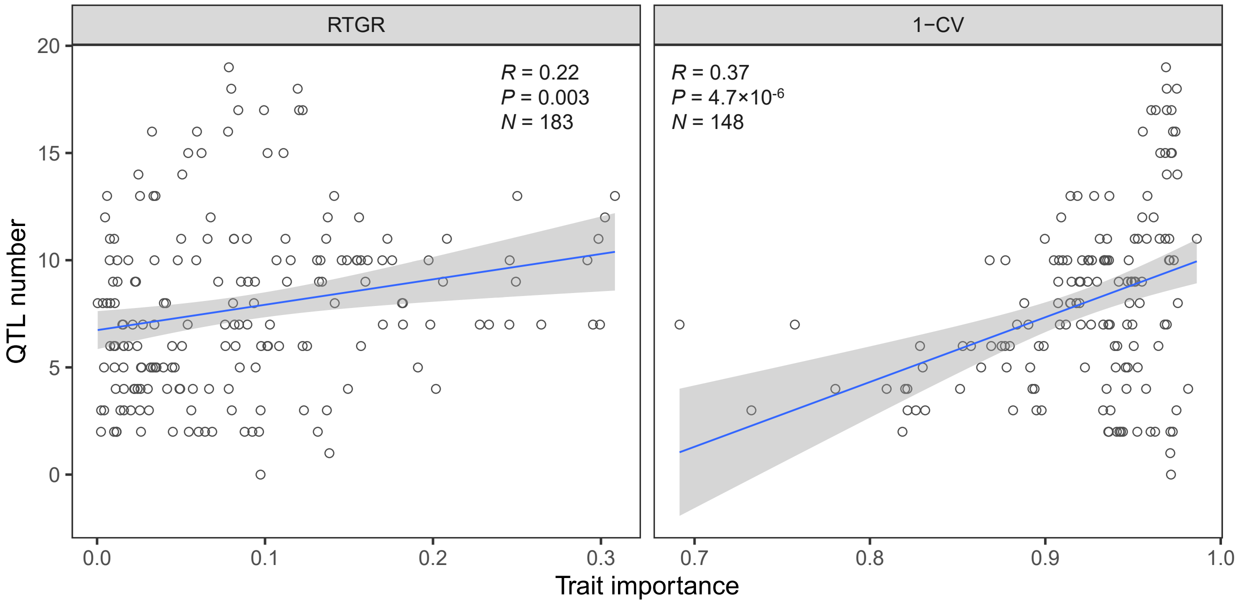

Figure S7. The positive correlation between QTL number and trait importance measured by RTGR (left) or 1-CV (right) largely remains when only traits with available f_{gene} values were considered. The number of traits with f_{gene} value are 183 and 148 for the panels of RTGR and 1-CV, respectively.

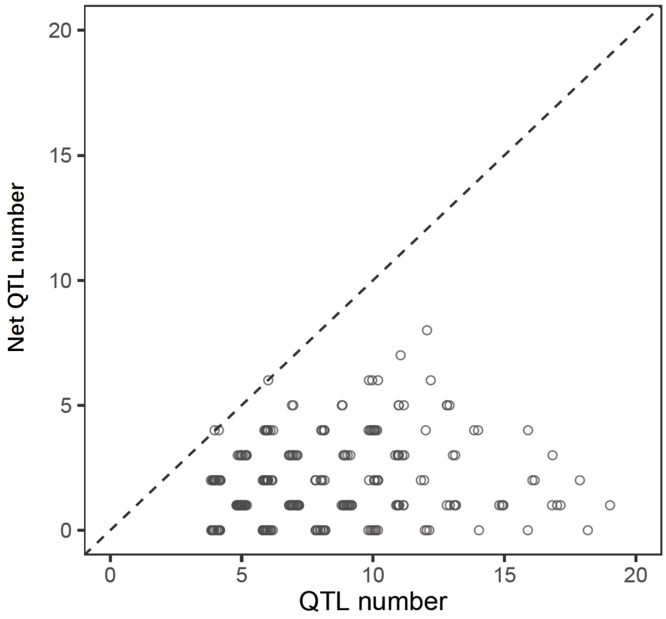

Figure S8. Analysis of the net QTL number to gauge the summed effects of multiple QTLs of a trait in a parental genome. The results suggest the phenotypic divergence between the BY and RM parents does not represent well the underlying genetic divergence. The net QTL number of a trait is defined by the absolute difference between the numbers of QTLs with opposite effects on the trait in a parent. Each circle represents a trait and a total of 294 traits each with at least four QTLs are included.

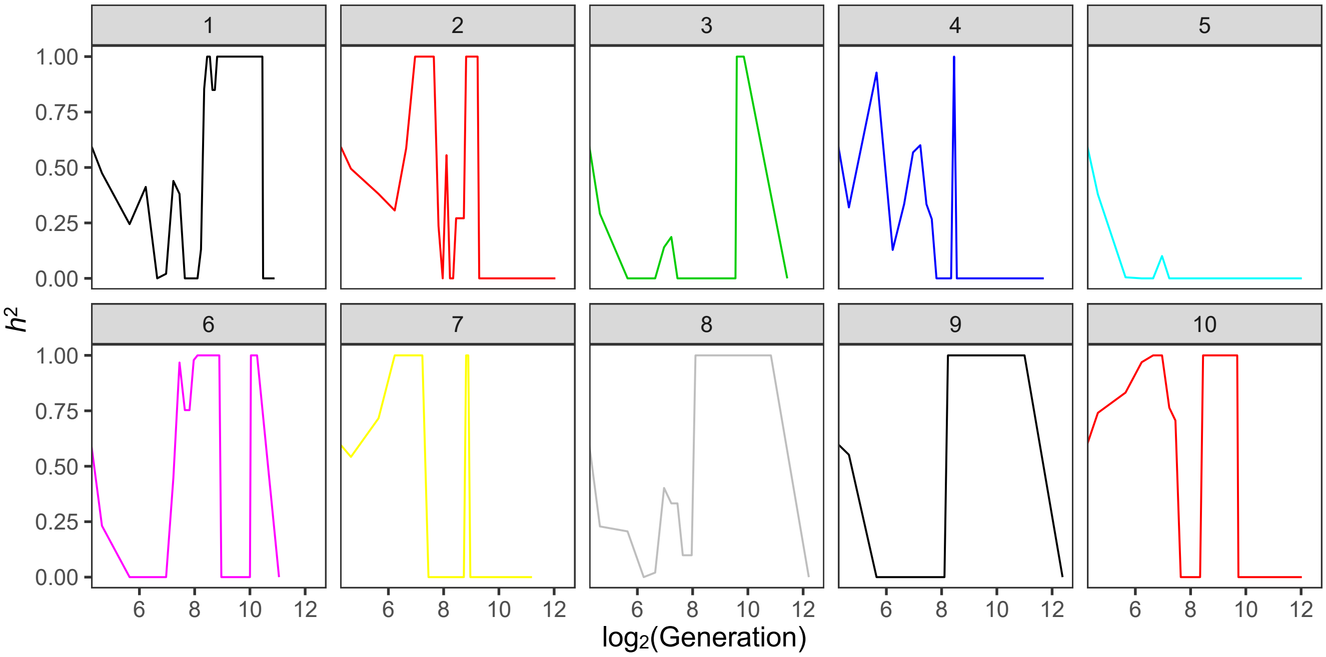

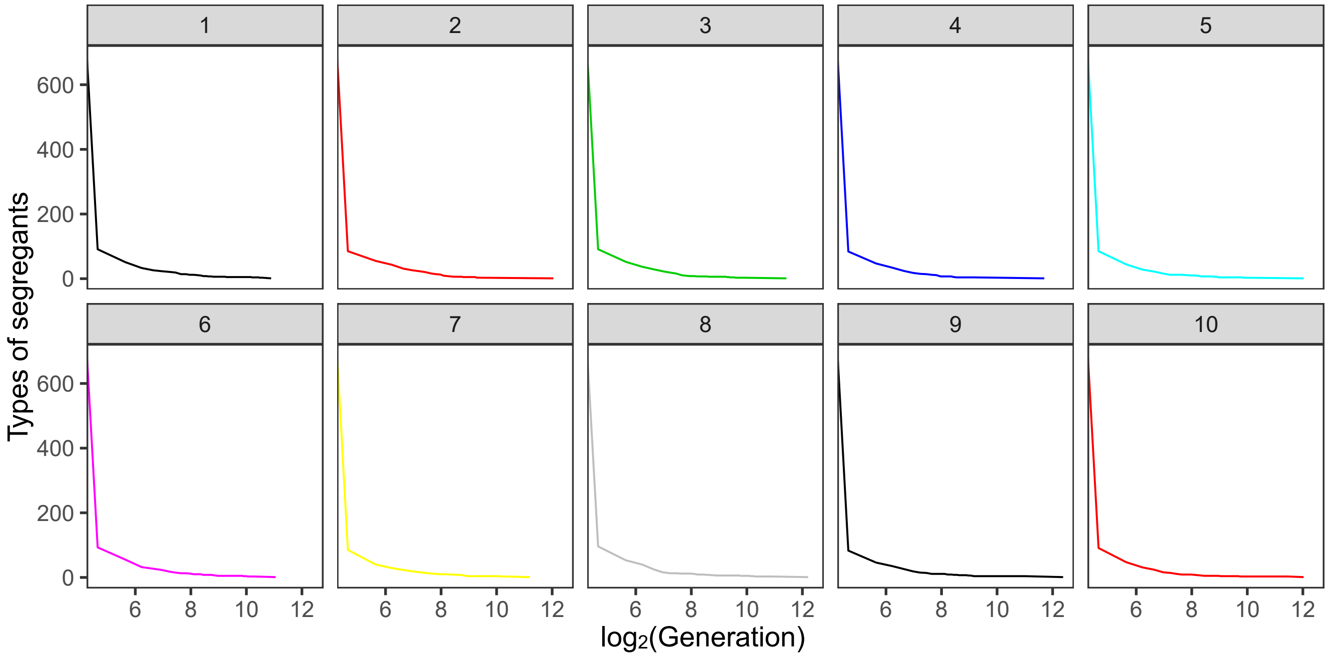

Figure S9: The evolutionary dynamics of h^2 of the trait D106_C in the yeast segregant population was simulated under the Wright-Fisher model with natural selection with ten replications. The dynamics of h^2 (upper panels) with the corresponding remaining segregant types (lower panesl) are shown as a function of evolutionary generation. As expected, the number of unique segregant types in the population decreases rapidly during the evolution. However, h^2 appears to show strong fluctuations even within a single replicate, likely due to the fact that V_P and V_A both reduce but at different speeds.To conduct the simulation, a total of 687 segregants with both the trait information and measured growth rate (as fitness) were used as the initial population. The evolution of this asexual population was then simulated under Wright-Fisher model with a constant population size (=687). For each replicate the simulation ended when only one unique type of segregant remained in the population.The number of remaining segregant types and h^2 of the trait in the population were calculated every 25 generations.
